# The consequences of niche and physiological differentiation of archaeal and bacterial ammonia oxidisers for nitrous oxide emissions

**DOI:** 10.1038/s41396-017-0025-5

**Published:** 2018-01-31

**Authors:** Linda Hink, Cécile Gubry-Rangin, Graeme W. Nicol, James I. Prosser

**Affiliations:** 1 School of Biological Sciences, University of Aberdeen, Cruickshank Building, Aberdeen, AB24 3UU UK; 2 Laboratoire Ampère, École Centrale de Lyon, Université de Lyon, Ecully, 69134 France; 3 Present Address: Laboratoire Ampère, École Centrale de Lyon, Université de Lyon, Ecully, 69134 France

**Keywords:** Microbiology, Ecology

## Abstract

High and low rates of ammonium supply are believed to favour ammonia-oxidising bacteria (AOB) and archaea (AOA), respectively. Although their contrasting affinities for ammonium are suggested to account for these differences, the influence of ammonia concentration on AOA and AOB has not been tested under environmental conditions. In addition, while both AOB and AOA contribute to nitrous oxide (N_2_O) emissions from soil, N_2_O yields (N_2_O–N produced per NO_2_^−^–N generated from ammonia oxidation) of AOA are lower, suggesting lower emissions when AOA dominate ammonia oxidation. This study tested the hypothesis that ammonium supplied continuously at low rates is preferentially oxidised by AOA, with lower N_2_O yield than expected for AOB-dominated processes. Soil microcosms were supplied with water, urea or a slow release, urea-based fertiliser and 1-octyne (inhibiting only AOB) was applied to distinguish AOA and AOB activity and associated N_2_O production. Low ammonium supply, from mineralisation of organic matter, or of the fertiliser, led to growth, ammonia oxidation and N_2_O production by AOA only, with low N_2_O yield. High ammonium supply, from free urea within the fertiliser or after urea addition, led to growth of both groups, but AOB-dominated ammonia oxidation was associated with twofold greater N_2_O yield than that dominated by AOA. This study therefore demonstrates growth of both AOA and AOB at high ammonium concentration, confirms AOA dominance during low ammonium supply and suggests that slow release or organic fertilisers potentially mitigate N_2_O emissions through differences in niche specialisation and N_2_O production mechanisms in AOA and AOB.

## Introduction

Microbes play central roles in global biogeochemical cycles but, despite evidence for niche differentiation, it is often difficult to identify and quantity the consequences of microbial community composition for rates of biogeochemical processes. Microbes are particularly important in the terrestrial nitrogen cycle, where they are solely responsible for many of the key processes, including nitrification, the oxidation of ammonia (NH_3_), via nitrite (NO_2_^−^), to nitrate (NO_3_^−^). NH_3_ oxidation is accompanied by production of nitrous oxide (N_2_O), an important greenhouse gas, with 300-fold and 20-fold greater global warming potentials than carbon dioxide and methane, respectively [1], while mono-nitrogen oxides, formed from N_2_O, contribute to depletion of stratospheric ozone [2]. N_2_O emissions following N-fertilisation of agricultural soils dominate N_2_O production in terrestrial environments and are predicted to increase with increased fertiliser demands [3]. Nitrification also leads to substantial loss of nitrogen fertilisers, through leaching of NO_3_^−^ [4] and sequential reduction of NO_3_^−^ by denitrifiers to NO_2_^−^, nitric oxide (NO), N_2_O and/or N_2_ [5].

There is some evidence for niche differentiation between archaeal and bacterial NH_3_ oxidisers (AOA and AOB), which are major players in soil NH_3_ oxidation [6, 7]. For example, the existence of obligately acidophilic AOA [8, 9], but not AOB, explains the global dominance of AOA in acid soils [10]. There is also evidence for a differential effect of ammonium (NH_4_^+^), with AOA rather than AOB being favoured in ‘low NH_4_^+^’, acidic or unfertilised soils [11–17, 49] and AOB being favoured in soils treated with high levels of NH_4_^+^-based fertiliser [14, 17, 18]. Attempts have been made to explain this by higher affinity for NH_3_ in AOA, based on reported lower *K*_m_ values (0.13–0.69 µM total ammonia nitrogen (TAN, NH_3_ + NH_4_^+^) for cultured marine and soil AOA [19–21] than those for AOB, which are 2–4 orders of magnitude higher [22–24]. While this provides a compelling explanation for numerical dominance of AOA in oceans, where TAN concentrations are in the nM range, it is less convincing in soil where bulk TAN concentrations are above the range of *K*_m_ values for both AOA and AOB. In addition, Hink et al. [25] and Kits et al. [26] report similar *K*_m_ values for AOB (*Nitrosomonas europaea*), *Nitrosopumilus maritimus* and other AOA and differences in substrate affinity do not explain niche differentiation in fertilised soil, where NH_3_ will be in excess. An alternative explanation is greater sensitivity of AOA to inhibition by high NH_4_^+^ concentration, based on studies of relatively few cultured AOA, but the recently isolated and enriched AOA *Candidatus* Nitrosocosmicus species [27–29] can grow at NH_3_ concentrations that inhibit other cultured AOA, with *Candidatus* Nitrosocosmicus franklandus growing at up to 100 mM NH_4_^+^, suggesting that NH_3_ toxicity does not clearly differentiate AOA and AOB. Soil microcosm studies also suggest greater complexity in the relationships between NH_3_ concentration, supply rate and AOA and AOB abundance and activity. For example, AOA can grow in soil amended with 0, 20 or 200 µg NH_4_^+^–N g^−1^ soil [18], can dominate oxidation of NH_3_ derived from mineralisation of native or added organic nitrogen and may not be stimulated by addition of inorganic NH_4_^+^ [49, 30]. These findings therefore suggest that AOB will dominate NH_3_ oxidation after addition of N-fertiliser at high concentration, while AOA will dominate when NH_4_^+^ is supplied at low rates, through mineralisation of organic or of slow-release fertilisers.

Niche differentiation of AOA and AOB associated with NH_4_^+^ supply has the potential to influence, significantly, N_2_O emissions due to their apparently distinct physiological processes. AOB produce N_2_O enzymatically via conversion of hydroxylamine (an intermediate in NH_3_ oxidation) to N_2_O via NO, rather than NO_2_^−^ (incomplete hydroxylamine oxidation) [31, 32], and via nitrifier denitrification, the sequential reduction of NO_2_^−^ to NO and N_2_O [32, 33]. In contrast, there is no genomic or physiological evidence for enzymatic production of N_2_O by AOA and NH_3_ oxidation-associated N_2_O emission is believed to result from an abiotic reaction between hydroxylamine and NO or NO_2_^−^ [34, 35]. These physiological differences are consistent with measured N_2_O yields (N_2_O–N produced per NO^2^–N generated from NH_3_ oxidation), with those from AOA cultures (0.004–0.23%) at the lower end of the range of those from AOB cultures (0.1–1%) [35–40]. This is also consistent with the low N_2_O yield associated with NH_3_ oxidation by AOA in an agricultural soil (~0.5‰), approximately half that of AOB [17]. These results therefore suggest that fertilisation strategies stimulating AOA rather than AOB would lead to lower N_2_O emissions in agricultural soils.

## Material and methods

### Soil microcosms

Soil microcosms were constructed as described in Hink et al. [17]. Briefly, soil was sampled from the upper 10 cm of a pH 6.5 sandy loam agricultural soil (SRUC, Craibstone, Scotland; grid reference NJ872104) with an organic C content of 5.9–6.4% (for soil characteristics see Kemp et al. [41] and Bartram et al. [42]) before sieving (3.35-mm mesh size) and storage at 4 °C before use. Water content was determined by drying the soil at 100°C for 24 h and microcosms were established in 120-ml serum bottles filled with 13.6 ± 0.02 g fresh soil (10 g dry soil, 27% volumetric water content). Bottles were sealed with butyl rubber stoppers and aluminium caps and pre-incubated at 30 °C in the dark for 7 days. Aerobic conditions were maintained by opening and re-sealing bottles after 4 days.

Following pre-incubation, microcosms were incubated at 30 °C in the dark, in the presence and absence of nitrification inhibitors (see below) with amendments designed to provide a single supply of NH_4_^+^ at high concentration or continuous supply of NH_4_^+^ at a low concentration during each of two phases of incubation (Fig. S1). NH_4_^+^ was supplied through mineralisation of native organic nitrogen and, in some treatments, by additional mineralisation of urea or of a slow-release, urea-based fertiliser (Azolon, Aglukon, Düsseldorf, Germany) that contains 15% free urea and 85% polymethylene urea. Free urea was converted to NH_4_^+^ within 8 h by ureolytic soil microorganisms, while urea was released at a low rate by mineralisation of polymethylene urea.

Mineralisation of native organic nitrogen occurred in all treatments and was the sole source of NH_4_^+^ in controls (addition of water only). In all fertiliser treatments, NH_4_^+^ was also produced by mineralisation, at a similar rate, of polymethylene urea. Both of these processes continued throughout incubation (for 24 days). Fertiliser addition at day 0 additionally led to a high initial concentration of NH_4_^+^, through mineralisation of free urea in the fertiliser, which was oxidised to NO_3_^−^ within ~10 days (phase 1), determined in preliminary experiments. A second, single supply of high NH_4_^+^ was achieved by addition of free urea to previously fertilised (and non-inhibited) treatments at the end of phase 1. This was rapidly converted to NH_4_^+^ and oxidised within the first 7 days of phase 2 (days 10–24).

Thus, at the beginning of phase 1, microcosms were amended with 0.5 ml water or 0.5 ml 1:100-diluted fertiliser. The final volumetric water content in all microcosms was 29% (equivalent to ~60% water-filled pore space (WFPS)). At the beginning of phase 2, microcosms were amended with 0.3 ml water or 0.3 ml urea solution (50 µg N g^−1^ soil_dw_) resulting in a volumetric water content of 30%.

Microcosms were also incubated in the presence or absence of the NH_3_ oxidiser inhibitors acetylene (inhibitory for both AOA and AOB) [12] and 1-octyne (inhibitory for AOB but not AOA) [17, 43]. This enabled differentiation between NH_3_ oxidation-related and non-NH_3_ oxidation-related processes, particularly N_2_O production, and between AOA and AOB activity and associated N_2_O production. Inhibitors were applied by injection into the headspace, with three treatments: air (no inhibitor), acetylene (0.1% v/v) or 1-octyne (0.03% v/v).

Each treatment was performed in triplicate. At each sampling point (at least twice weekly), gas samples (5 ml) were taken from each microcosm and transferred into evacuated 3-ml glass vials (Labco, Lampeter, UK) for subsequent N_2_O analysis. Microcosms were destructively sampled and soil was stored immediately after sampling at −20 °C for further chemical (storage ≤ 2 weeks) and molecular analysis (storage ≤ 4 months). Oxic conditions in the remaining microcosms were maintained by opening and re-sealing twice weekly, while re-establishing concentrations of acetylene or 1-octyne where appropriate.

### Chemical analysis of soil and gas samples

Due to the rapid conversion of urea to NH_4_^+^, nitrification kinetics were determined as temporal changes in concentrations of NH_4_^+^, NO_2_^−^ and NO_3_^−^, which were measured colorimetrically in 96-well microplates following KCl (1 M) extraction (1:5 soil:KCl ratio (v/v)). Ammonium was determined using the indophenol method [44] as described previously [8], with a detection limit of 20 µM. The measurement of NO_2_^−^ and NO_3_^−^ was modified from Shinn [45], and Doane and Horwath [46] as follows: NO_2_^−^was measured in a 50-µl sample by sequentially adding 60 µl of diazotising reagent (2.2 mM sulphanilamide in 3.3 M HCl) and 20 µl coupling reagent (0.12 mM N-(1-naphthyl)-ethylenediamine in 0.12 M HCl). NO_2_^−^ and combined NO_2_^−^ and NO_3_^−^ were determined before and after reduction of NO_3_^−^ to NO_2_^−^ by addition of 20 µl vanadium chloride solution (4.5 mM vanadium(III) chloride in 1 M HCl) and incubation in the dark at 35 °C for 90 min, respectively. NO_2_^−^ was below the detection level (2.5 µM) in all samples and nitrification kinetics were determined as rates of NH_3_ oxidation or NO_3_^−^ production. Soil pH was measured in water (1.5 g wet soil + 3 ml deionised water). N_2_O in the headspace samples was determined with an Agilent 6890 gas chromatograph equipped with a ^63^Ni electron capture detector (Santa Clara, CA, USA), as described in Hink et al. [17].

### Nucleic acid extraction and estimation of ammonia oxidiser abundance

Nucleic acids were extracted from 0.5 g wet soil as described previously by Nicol and Prosser [47]. The abundances of AOA and AOB were assessed by qPCR of the respective ammonia monooxygenase subunit A (*amoA*) gene in all samples except those treated with acetylene, as previous studies using this soil (e.g. [12, 17]) have demonstrated complete inhibition of AOA and AOB growth and activity using the same or lower concentrations of acetylene. NH_3_ oxidiser growth was assessed by quantification of AOA and AOB *amoA* genes as described previously [48] with *r*^2^ values > 0.99 and amplification efficiencies of 95–99% and 88–95% for archaeal and bacterial assays, respectively.

### Statistical analysis

Statistical analysis was performed using R 3.3.3 (http://www.rproject.org/). Temporal differences in NH_4_^+^, NO_3_^−^ and N_2_O concentrations were assessed by ANOVA and comparison of the slopes of the linear models with fertiliser and inhibitor treatment as categorical factors and time as a continuous factor. N_2_O accumulation was analysed by fitting the regression to the cumulative data over time (estimated using all possible combinations of N_2_O accumulated in 3−4-day intervals of the destructively sampled replicates over the whole incubation period). Differences between N_2_O yield of AOA, AOB and combined AOA and AOB were determined by ANOVA, followed by a Tukey *post hoc* test. Differences in *amoA* gene abundance between treatments were assessed independently within phase 1 and phase 2 (due to an unbalanced design) by investigating the overlapping of 95% confidence intervals of linear regressions fitted to log_10_ transformed temporal changes in AOA *amoA* gene abundance (log_10_ y = ax + b) and quadratic polynomial regressions fitted to temporal changes in AOB *amoA* gene abundance (y = y_0_ + ax + bx^2^).

## Results

### Nitrification, N_2_O accumulation and ammonia oxidiser growth at low NH_4_^+^ supply

**Fig. 1:**
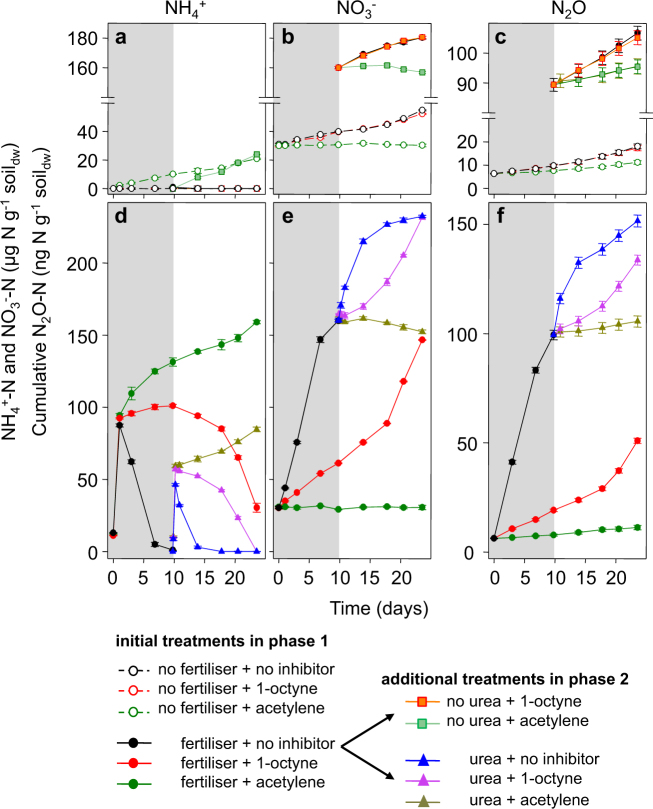
Changes in NH_4_^+^, NO_3_^−^ and N_2_O during incubation of soil microcosms for 24 days. Microcosms were incubated after amendment with a slow-release, urea-based fertiliser that contained 15% free urea, or with water only (no fertiliser), in combination with 1-octyne, acetylene or no inhibitor. **a**–**c** present data in which NH_4_^+^ was supplied at a low continuous rate, through slow mineralisation of native organic nitrogen (phases 1 and 2) or of polymethylene urea (phase 2). **d**–**f** present data in which NH_4_^+^ was supplied at a single high concentration, through rapid mineralisation of free urea within the slow-release fertiliser (phase 1) or through addition of urea (phase 2). Phase 1 (days 0–10) is indicated by a grey background and phase 2 (days 10–24) by a white background. Inhibitor treatments were applied to fertiliser-amended microcosms by additional amendment with urea or water. NH_4_^+^ and NO_3_^−^ concentrations were determined in destructively sampled microcosms and cumulative N_2_O production was determined following repeated sampling of headspace gas. Data represent mean values and standard errors of three replicate microcosms

Nitrification in control microcosms was driven in both phases 1 and 2 by mineralisation of native organic nitrogen. Mineralisation rate during phase 1 was ~1 µg NH_4_^+^ g^−1^ soil_dw_ d^−1^, estimated as the increase in NH_4_^+^ in acetylene-amended control microcosms, in which both AOA and AOB were inhibited and NO_3_^−^ production was negligible (Fig. 1a, b). A small increase in N_2_O (~6 ng N_2_O-N g^−1^ soil_dw_ after 24 days) was also observed in acetylene-treated microcosms (Fig. 1c).

**Fig. 2:**
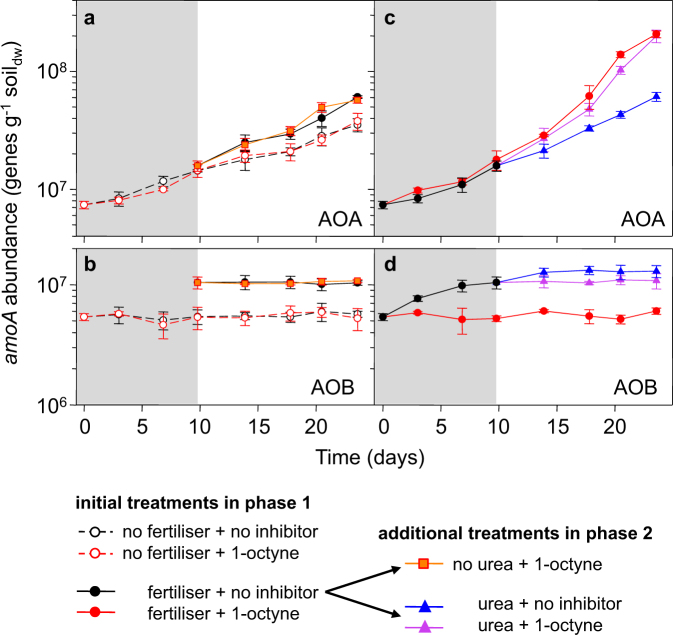
Changes in abundance of archaeal and bacterial *amoA* genes during incubation of soil microcosms for 24 days. Quantification was performed on extracted DNA from destructively sampled soil microcosms that were amended with fertiliser or water only (no fertiliser) in combination with 1-octyne or no inhibitor. **a**, **b** present data in which NH_4_^+^ was supplied at a low continuous rate, through slow mineralisation of native organic nitrogen (phases 1 and 2) or of polymethylene urea (phase 2). **c**, **d** present data in which NH_4_^+^ was supplied at a single high concentration, through rapid mineralisation of free urea within the slow-release fertiliser (phase 1) or through addition of urea (phase 2). Representation of phases 1 and 2 and treatments are as described in the legend for Fig. 1. Mean concentrations and standard errors of triplicate microcosms are plotted. Differences in temporal changes were assessed by comparing confidence intervals of regression analysis (see Figs. S2 and S3)

In non-inhibited control microcosms, NO_3_^−^ was produced at a rate similar to the mineralisation rate (~1 µg NO_3_^−^ g^−1^ soil_dw_ d^−1^) and NH_4_^+^ was below the minimum detection level throughout incubation (Fig. 1a, b). NH_3_ oxidation in these microcosms increased N_2_O production by ~7 ng N_2_O-N g^−1^ soil_dw_ (Fig. 1c). NO_3_^−^ and N_2_O production rates in 1-octyne-inhibited and non-inhibited microcosms were not significantly different, suggesting that oxidation of NH_3_ derived from mineralisation of native organic nitrogen was performed by AOA and not AOB. This was confirmed by analysis of temporal changes in AOA and AOB abundances. AOA grew in control microcosms throughout incubation for 24 days and specific growth rates were similar in non-inhibited and 1-octyne-treated microcosms (Fig. 2a, Fig. S2a). AOB *amoA* gene abundance, however, did not change during incubation in the presence or absence of 1-octyne (Fig. 2c; Fig. S3a). Nitrification activity was accompanied in these microcosms by a slight decrease in pH, from 6.6 to 6.5 due to proton release associated with NH_3_ oxidation, as commonly observed (Fig. S4).

NH_4_^+^ was also supplied at low rates in fertilised microcosms during phase 2, after consumption, during phase 1, of NH_4_^+^ initially present at high concentration following conversion of free urea. These microcosms were incubated with or without addition of acetylene or 1-octyne in phase 2. Addition of acetylene enabled estimation of the rate of production of NH_4_^+^ in phase 2, derived from both slow release of urea and mineralisation of native organic nitrogen. This rate, ~2 µg NH_4_^+^ g^−1^ soil_dw_ d^−1^ (Fig. 1a), was approximately twice the rate of production from mineralisation of native organic nitrogen alone (*P* < 0.001). NO_3_^−^ decreased slightly in these microcosms by ~5 µg NO_3_^−^ g^−1^ soil_dw_ (Fig. 1b) and pH decreased from 6.1 to 6.0 (Fig. S4). The decrease in NO_3_^−^ may have resulted from immobilisation or reduction of NO_3_^−^, although potential N_2_O emission due to denitrification was negligible, as N_2_O accumulation in acetylene-treated microcosms was generally very low and not significantly different from accumulation in control microcosms (*P* > 0.05). The rate of NH_4_^+^ supply was confirmed by measurement of NO_3_^−^ production in non-inhibited microcosms, where NH_4_^+^ was stoichiometrically converted to NO_3_^−^ and differences in NO_3_^−^ concentration between the acetylene-treated and non-inhibited microcosms reflected the mineralisation rate (~2 µg NO_3_^−^ g^−1^ soil_dw_ d^−1^; Fig. 1b; *P* < 0.001).

AOA growth was greater in fertilised, 1-octyne-inhibited microcosms during phase 2 than in fertilised, non-inhibited microcosms during phase 1 (Fig. 2a) and was greater in both of these treatments than in unfertilised microcosms during phase 2 (Fig. 2a, Fig. S2b). 1-octyne prevented growth of AOB (Fig. 2b) and NO_3_^−^ production (Fig. 1b), and therefore N_2_O accumulation (10 ng N_2_O–N g^−1^ soil_dw_; Fig. 1c) and N_2_O production were associated with archaeal, rather than bacterial NH_3_ oxidation in microcosms in which NH_4_^+^ was supplied at a continuous low rate.

### Nitrification, N_2_O accumulation and ammonia oxidiser growth at high NH_4_^+^ supply

In fertilised microcosms, NH_4_^+^ concentration increased to ~13 µg NH_4_^+^ g^−1^ soil_dw_ immediately after amendment at the beginning of phase 1, through rapid conversion of free urea contained in the fertiliser, and reached ~90 µg NH_4_^+^ g^−1^ soil_dw_ after 1 day of incubation (Fig. 1d). In microcosms additionally treated with acetylene, NH_4_^+^ concentration continued to increase throughout incubation, due to mineralisation of native organic nitrogen and additional supply through slow release of urea, following mineralisation of polymethylene urea. There was no detectable increase in NO_3_^−^ concentration in the presence of acetylene (Fig. 1e; *P* < 0.05), but a slight increase in N_2_O accumulation that was similar to that in acetylene-treated control microcosms (Fig. 1f; *P* > 0.05). In the absence of inhibitors, NH_4_^+^ was quickly and stoichiometrically converted to NO_3_^−^, at a rate of ~17 µg N g^−1^ soil_dw_ d^−1^, estimated during the first week of incubation. This rate then decreased until NH_4_^+^ became undetectable, after incubation for 10 days (Fig. 1d and e). N_2_O accumulation followed similar kinetics, with an initial high rate of ~11 ng N_2_O-N g^−1^ soil_dw_ d^−1^ (Fig. 1f).

NH_3_ oxidation was accompanied by significant growth of AOB, which ceased as NH_4_^+^ was depleted (Fig. 2d; Fig. S3a). AOA *amoA* gene abundance also increased during phase 1, at high NH_4_^+^ concentration, at similar rates in the absence and presence of 1-octyne (Fig. 2c), and abundances were significantly greater than those at low NH_4_^+^ supply (Fig. S2a). N_2_O accumulation at high concentrations of NH_4_^+^ therefore resulted from the activities of both AOA and AOB. In the presence of 1-octyne, initial nitrification and N_2_O accumulation rates were reduced by ~80% and 90%, respectively, indicating that AOB dominated NH_3_ oxidation and associated N_2_O production. 1-octyne application inhibited AOB growth (Fig. 2d) and NH_4_^+^ concentration remained high throughout phase 2 (Fig. 1e), leading to greater stimulation of AOA growth than in microcosms with lower NH_4_^+^ concentration (Fig. 1a, b; Fig. S2b). NH_3_ oxidation in phase 1 decreased soil pH from ~6.9 to ~6.1, which was counterbalanced by an initial increase of ~0.3 units of pH following fertiliser addition, leading to an overall difference between the pH of fertilised and unfertilised soil of only ~0.5 at day 10 (Fig. S4).

NH_4_^+^ was also supplied at high concentration following rapid conversion (within 8 h) of urea added at the beginning of phase 2, in the presence or absence of inhibitors. This was followed by a slower increase in NH_4_^+^ concentration in acetylene-treated microcosms due to production of NH_4_^+^ from native organic nitrogen and the slow-release fertiliser (Fig. 1c). In non-inhibited, urea-amended microcosms, NH_4_^+^ was stoichiometrically converted to NO_3_^−^ within 4 days and initial nitrification rates and N_2_O kinetics were similar to those in phase 1 in non-inhibited, fertilised microcosms. This implies that AOB were not affected by the decrease in pH during phase 1. The initial high rates were likely associated with AOB growth, but AOB *amoA* gene abundances were not significantly different from those in the treatment with no urea added (Fig. S3b).

AOA growth was similar to that when NH_4_^+^ was derived from slow release of urea, but was stimulated more after addition of 1-octyne (Fig. 2c, Fig. S2b). However, this growth was not significantly different to that estimated when NH_4_^+^ concentration was high throughout incubation in microcosms amended with fertiliser in the presence of 1-octyne from the beginning of incubation (Fig. S2b).

Together these data indicate that both AOA and AOB are capable of NH_3_ oxidation, growth and N_2_O production at high concentrations of NH_4_^+^ but that, in this soil, the contribution of AOB is greater. In addition, growth and activity of AOA dominated when NH_4_^+^ became undetectable and when 1-octyne was present at high NH_4_^+^ concentrations, which led to greater stimulation of growth, likely due to the removal of competition with AOB for substrate.

### N_2_O yield of AOA and AOB

**Fig. 3:**
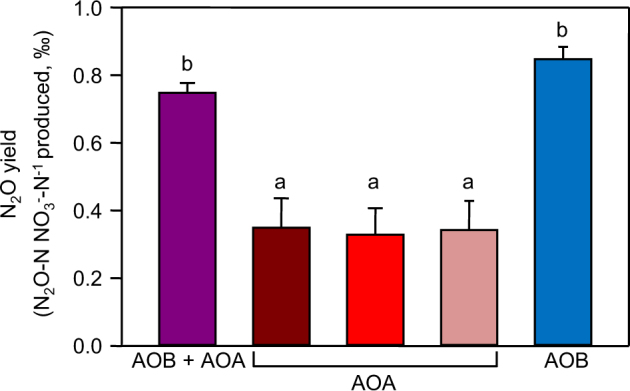
The yield of N_2_O associated with ammonia oxidation by AOA and AOB. N_2_O yield associated with activity of both AOA and AOB at high NH_4_^+^ concentration in fertilised microcosms during phase 1 (purple bar); AOA only oxidising NH_3_ derived from mineralisation of native organic nitrogen (dark red bar), from mineralisation of native organic nitrogen and from slowly released urea during phase 2 (medium red bar) or from mineralisation of free fertiliser-urea with inhibition of AOB by 1-octyne during phases 1 and 2 (light red bar); AOB only, calculated based on the known yield of AOA and both AOA and AOB, in addition to the observation that under conditions where both were contributing to NH_3_ oxidation, ~80% was performed by AOB (blue bar). Mean yields and standard errors are plotted. Significant differences are indicated by different lower case letters

N_2_O yield was determined as the amount of N_2_O produced per NO_3_^−^ produced and was used to relate nitrification activity and N_2_O production by AOA and AOB (Fig. 3). To assess N_2_O production associated with NH_3_ oxidation only, non-ammonia oxidiser N_2_O produced in acetylene-treated microcosms was subtracted from that produced in those in which NH_3_ was oxidised. Only AOA were responsible for the N_2_O emission associated with the consumption of NH_4_^+^ derived from native organic nitrogen, from slow release of the fertiliser in phase 2 and from high NH_4_^+^ concentration in the presence of the AOB inhibitor, 1-octyne. In all of these situations, N_2_O yield was similar at ~0.35‰. Both AOB and AOA were responsible for consumption of NH_4_^+^, initially at high concentration, derived from free urea contained in the fertiliser in phase 1, during which N_2_O yield was ~0.75‰. As AOB were responsible for ~80% of the NH_3_ oxidation under these conditions, the AOB N_2_O yield was calculated to be ~0.85‰.

## Discussion

### Niche specialisation of AOA and AOB associated with NH_4_^+^ supply and concentration

A major aim of this study was to test the hypothesis that AOA and AOB dominate NH_3_ oxidation under conditions of low and high NH_4_^+^ supply, respectively. Evidence for this arose from correlations of AOA and AOB relative abundances in soils subjected to different fertiliser regimes [14–17], lack of stimulation of NH_3_ oxidation by supply of relatively high concentrations of inorganic NH_4_^+^, but stimulation by organic nitrogen in AOA-dominated soils [30, 49], and higher relative abundances of AOA where NH_4_^+^ is typically derived from mineralisation of native organic nitrogen [11, 30]. Detailed analysis of the effects of NH_4_^+^ supply and concentration on competition between AOA and AOB was not possible in previous studies, but was enabled here by the controlled supply of NH_4_^+^ through use of a slow-release fertiliser or addition of urea, use of inhibitors of both AOA and AOB and a specific inhibitor of AOB, and simultaneous measurement of NH_4_^+^ concentration and AOA and AOB abundances. Competition is used, here, in terms of its effect on cell yield rather than specific growth rate. Both AOA and AOB will be expected to grow at their maximum specific rates when NH_3_ concentration is high, but cell yield of both AOA and AOB will be reduced when both are utilising NH_3_.

This study provides strong evidence that AOA successfully outcompete AOB in soil in which NH_4_^+^ is continuously supplied at a low rate. This was achieved through mineralisation of native organic nitrogen or through additional supply using a slow-release fertiliser (phase 2), after oxidation of NH_4_^+^ derived from free urea that is also present in this fertiliser (phase 1). Mineralisation rate (~1 µg N g soil_dw_ d^−1^) was similar to those observed in previous studies of this soil at near neutral or neutral pH, which also indicated preferential activity of AOA [12, 13, 17]. AOA are also >10-fold more abundant than AOB in other soils with low NH_4_^+^ supply from mineralisation [11, 14–16, 18, 49]. AOA abundance increased but AOB abundance did not change significantly and nitrification rates were identical in the presence and absence of 1-octyne, which reduces nitrification strongly in AOB-dominated soils due to inactivation of AOB ammonia monooxygenase, as indicated by inhibition of activity, growth and transcriptional activity of AOB [16, 17, 43, 50]. Slow release of urea in phase 2 of this study doubled the NH_4_^+^ supply rate to ~2 µg N g soil_dw_ d^−1^ and increased AOA growth and nitrification rate, again with no detectable AOB growth or activity.

Reduction in NH_4_^+^ concentration to undetectable levels demonstrates that potential NH_3_ oxidation rate is greater than mineralisation rate in this soil and the simplest explanation for growth of AOA and not AOB is that AOA have significantly greater affinity for NH_3_ than AOB. This is consistent with reported higher *K*_m_ values for several AOB cultures (27–825 µM TAN) [20, 51, 52] than pure (*N. maritimus*) and enrichment cultures of AOA (0.13–0.69 µM TAN) [19–21]. Recent determination of similar *K*_m_ values of *N. europaea* and *N. maritimus* [25] and of other AOA [26] questions the strength of this argument. In addition, this proposed explanation does not consider the complexity of the soil environment, where NH_4_^+^ is adsorbed to clay minerals and supply of NH_4_^+^ produced from mineralisation of organic nitrogen or urea requires diffusion through a spatially heterogeneous environment. Moreover, *K*_m_ values have not been determined for typical soil AOA or AOB and may not reflect those of natural communities, while soil NH_4_^+^ concentrations are generally well above the nM *K*_m_ values previously reported for AOA.

AOB growth occurred only when NH_4_^+^ supply exceeded potential NH_3_ oxidation rate when NH_4_^+^ was formed from free urea, initially present in the fertiliser (phase 1) or urea added separately (phase 2). In both cases, AOB growth ceased when NH_4_^+^ became undetectable, whereas AOA growth continued. This finding is consistent with several studies reporting AOB growth or high relative abundance in soils with high NH_4_^+^ availability, such as those subjected to high levels of NH_4_^+^-based fertiliser [14, 17, 18, 53]. However, AOA also grew under these conditions, despite high NH_4_^+^ concentration. In phase 1, AOA growth was significantly greater than that with low NH_4_^+^ supply and in phase 2, AOA growth in microcosms with high NH_4_^+^ availability was significantly greater when AOB were inhibited by 1-octyne, suggesting direct competition between AOA and AOB for NH_3_. AOB did, however, dominate NH_3_ oxidation, contributing 80%, as determined by comparison of initial rates with and without specific inhibition of AOB.

Although dominance of AOB in heavily fertilised soils has been explained through differences in *K*_m_ values (see above), any effects on growth and activity at NH_4_^+^ concentrations significantly higher than *K*_m_ values will be negligible. The alternative explanation of greater sensitivity of AOA to high NH_4_^+^ concentrations is also not supported, as both AOA and AOB grew at moderately high NH_4_^+^ concentrations. The recent cultivation of three *Nitrosocosmicus* strains capable of growth at high NH_4_^+^ concentration [27–29] also suggests that this is not a general explanation for AOB dominance.

Changes in the activity and competitive ability of AOA may have resulted from changes in community composition, potentially selecting, for example, for NH_3_-tolerant phylotypes such as *N. franklandus* [28]. Hink et al. [17] did not observe changes in the transcriptionally active AOA community in response to high NH_4_^+^ supply and removal of competition with AOB (1-octyne application) during incubation of the same soil for 13 days, but detected changes after 20 days of incubation. Verhamme et al. [18] observed AOA growth and changes in community structure in soil that was unamended or repeatedly spiked with ‘intermediate’ and ‘high’ concentrations of NH_4_^+^ to maintain concentrations of 20 and 200 NH_4_^+^–N g soil_dw_. In contrast, significant growth of AOB was only detected at the highest concentration of added NH_4_^+^. Soil pH influences AOB and AOA growth, activity and community composition [6, 7] and NH_3_ oxidation is accompanied by the release of protons that can reduce soil pH. The largest pH change in the current study was from 6.9 to 6.1 during phase 1 incubation of fertilised microcosms. This, however, did not significantly influence AOB activity, which dominated in these microcosms, as nitrification rate was similar during the consumption of NH_4_^+^ in phase 1, derived from free urea in the fertiliser, and urea added in phase 2. There is no evidence, therefore, that reduction in soil pH resulted in dominance of AOA over AOB under conditions of low NH_4_^+^ supply.

These results provide evidence for the hypothesis that AOA are responsible for NH_3_ oxidation when NH_4_^+^ is supplied at a low rate. They also provide evidence that AOB outcompete AOA at high supply rate but AOA growth occurred at high NH_4_^+^ concentration and accelerated when AOB were inhibited. There is therefore evidence for niche differentiation associated with NH_4_^+^ supply rate but the precise underlying mechanism remains unclear.

### The consequences of NH_4_^+^ supply rate for N_2_O production and mitigation strategies

AOA and AOB were responsible for N_2_O production in this aerobic soil. Emissions from heterotrophic denitrifiers were unlikely or negligible, as N_2_O production was always associated with NH_3_ oxidation and NO_3_^−^ production, denitrification was excluded previously under similar experimental conditions [17] and denitrifier activity in this soil is very low at similar moisture content (WFPS ≤60%) [54]. Nevertheless, N_2_O accumulated slowly in acetylene-treated microcosms, when NH_3_ oxidiser growth and activity were inhibited. This was not further investigated, but a possible explanation is abiotic production, although such reactions are usually negligible when the intermediates nitrite and hydroxylamine are not provided by NH_3_ oxidisers [55–57].

Production of N_2_O and NO_3_^−^ was generally coordinate, but relative rates of production and, consequently, N_2_O yield were dependent on the organisms dominating NH_3_ oxidation. Under conditions of low NH_4_^+^ supply, when AOA dominated NH_3_ oxidation, or at high NH_4_^+^ supply, when AOB were inhibited, N_2_O yield was ~0.35‰, while at high NH_4_^+^ supply, when ~80% of NH_3_ oxidation was performed by AOB, N_2_O yield was ~0.75‰ and estimated as ~0.85‰ calculated for AOB only. These yields are similar to values of 0.5‰, and 0.9‰, for AOA and AOB, respectively, reported previously by Hink et al. [17]. They are also consistent with current understanding of mechanisms for N_2_O production in AOA, where production is limited to presumably abiotic hybrid formation [34, 35], and in AOB, which can additionally produce N_2_O through nitrifier denitrification and as a by-product of hydroxylamine metabolism [34]. Although NH_4_^+^ concentration may have led to selection, or selective activity of different AOA phylotypes, N_2_O yield did not differ significantly when AOA dominated at low NH_4_^+^ concentrations or at high NH_4_^+^ concentrations when AOB were specifically inhibited. Giguere et al. [50] obtained a relationship of increasing AOA-associated N_2_O production and accumulated NO_2_^−^, which is an intermediate during NH_3_ oxidation. However, NO_2_^−^ was below the detection limit in the study and there is no evidence of NO_2_^−^ accumulation during nitrification in Craibstone soil (e.g. [48]).

These results therefore demonstrate the consequences of niche specialisation in an important group of nitrogen-cycling organisms and consequences for rates of an important biogeochemical process. They also suggest that knowledge of NH_3_ oxidiser community composition can inform fertiliser strategies to optimise nitrogen fertiliser use efficiency, can minimise fertiliser loss and N_2_O emissions from terrestrial systems and can improve prediction of N_2_O emissions in climate change models. Current strategies to increase fertiliser use efficiency include application of nitrification (NH_3_ oxidiser) inhibitors [58], specific timing of fertiliser application [59, 60] and use of slow-release fertilisers [61–63]. Nitrification rate of inorganic fertiliser, supplied at high concentration, is lower in AOA-dominated soils (e.g. acidic soils) than those dominated by AOB, while nitrification rate in soil dominated by AOB is lower when NH_4_^+^ is derived from slow-release fertilisers or slowly degradable organic fertilisers. Coordinate NH_3_ oxidation and N_2_O production under aerobic conditions indicate that any strategy to reduce NH_3_ oxidation will mitigate N_2_O production, in addition to reducing production of N_2_O by denitrifiers at lower oxygen concentrations. However, strategies that increase dominance of NH_3_ oxidation by AOA (e.g. reduction in pH, development and use of specific AOB inhibitors) will decrease the proportion of oxidised NH_3_ that is converted into N_2_O. For example, long-term fertilisation with organic, rather than mineral nitrogen has been shown to increase AOA abundance [64] and, in a 3-year experiment, application of composted sludge or dried pellets reduced N_2_O emissions by >60% and slow-release fertiliser reduced emissions by 85%, in comparison with conventional mineral fertilisation [65]. Obviously, such strategies require consideration of potential effects on crop yield, in addition to feasibility and cost, but the study provides the basis for better informed development of fertilisation strategies and the potential to improve predictions of N_2_O emissions from terrestrial environments.

## Electronic supplementary material

Supplemental material
