# Supplementary material for: The consequences of niche and physiological differentiation of archaeal and bacterial ammonia oxidisers for nitrous oxide emissions
Source: ISME J. 2018 Jan 31;12(4):1084–93. doi: 10.1038/s41396-017-0025-5 (PMC5864188; doi:10.1038/s41396-017-0025-5)
Supplement: Supplementary file 1 — Supplemental material [file 41396_2017_25_MOESM1_ESM.docx]

**Supplementary material**

**Fig. S1.** Experimental design for microcosm studies. Phase 1 was initiated by addition of water (control microcosms) or fertiliser. Microcosms were incubated without nitrification inhibitor (no inhibitor), with acetylene (inhibition of AOA and AOB) or 1-octyne (inhibition of AOB only). While these treatments were maintained throughout the whole incubation period, additional treatments were established in phase 2, in which microcosms previously treated with fertiliser and no inhibitor were amended with either fertiliser or supplemented with urea in combination with and without inhibitors.

# Fig. S2. Linear regression fitted to log_10_ AOA *amoA* gene abundance during incubation of soil microcosms (see legend to Fig. 1 for details; A: phase 1, days 0 - 10; B: phase 2, days 10 - 24; colour of regressions and confidence intervals according to treatment). Solid and thin dashed lines represent regressions and confidence intervals for treatments with applied nitrogen, respectively, and thick dashed and dotted lines represent regressions and confidence intervals for treatments with nitrogen derived from mineralisation only, respectively.

#

# Fig. S3. Quadratic polynomial regression fitted to AOB *amoA* gene abundance during incubation of soil microcosms (see legend to Fig. 1 for details; A: phase 1, days 0 - 10; B: phase 2, days 10 - 24; colour of regressions and confidence intervals according to treatment). Solid and thin dashed lines represent regressions and confidence intervals for treatments with applied nitrogen, respectively, and thick dashed and dotted lines represent regressions and confidence intervals for treatments with nitrogen derived from mineralisation only, respectively.

**Fig. S4.** Changes in pH during incubation of soil microcosms for 24 days (see legend for Fig. 1 for details; phase 1: grey background, days 0 – 10; phase 2: white background, days 10 - 24). Mean pH and standard errors of triplicate microcosms are plotted.
